# Heterologous expression of high-activity cytochrome P450 in mammalian cells

**DOI:** 10.1038/s41598-020-71035-5

**Published:** 2020-08-25

**Authors:** Masaki Kumondai, Eiji Hishinuma, Evelyn Marie Gutiérrez Rico, Akio Ito, Yuya Nakanishi, Daisuke Saigusa, Noriyasu Hirasawa, Masahiro Hiratsuka

**Affiliations:** 1 Laboratory of Pharmacotherapy of Life-Style Related Diseases, Graduate School of Pharmaceutical Sciences, Tohoku University, 6-3 Aoba Aramaki, Aoba-ku, Sendai, 980-8578 Japan; 2 Advanced Research Center for Innovations in Next-Generation Medicine, Tohoku University, 2-1 Seiryo-machi, Aoba-ku, Sendai, 980-8575 Japan; 3 Tohoku Medical Megabank Organization, Tohoku University, 2-1 Seiryo-machi, Aoba-ku, Sendai, 980-8573 Japan; 4 Department of Pharmaceutical Sciences, Tohoku University Hospital, 1-1 Seiryo-machi, Aoba-ku, Sendai, 980-8574 Japan

**Keywords:** Biochemistry, Biological techniques, Cell biology

## Abstract

The evaluation of Cytochrome P450 (CYP) enzymatic activity is essential to estimate drug pharmacokinetics. Numerous CYP allelic variants have been identified; the functional characterisation of these variants is required for their application in precision medicine. Results from heterologous expression systems using mammalian cells can be integrated in in vivo studies; however, other systems such as *E. coli*, bacteria, yeast, and baculoviruses are generally used owing to the difficulty in expressing high CYP levels in mammalian cells. Here, by optimising transfection and supplementing conditions, we developed a heterologous expression system using 293FT cells to evaluate the enzymatic activities of three CYP isoforms (CYP1A2, CYP2C9, and CYP3A4). Moreover, we established co-expression with cytochrome P450 oxidoreductase and cytochrome b_5_. This expression system would be a potential complementary or beneficial alternative approach for the pharmacokinetic evaluation of clinically used and developing drugs in vitro.

## Introduction

Cytochrome P450 (CYP) is a superfamily of enzymes responsible for the biotransformation of most clinically used drugs^1^. Among the human CYP isoforms, the CYP1-3 subfamily is the most significant contributor to drug metabolism^2^. It is well known that gene variations implicated in drug pharmacokinetics as well as age, gender, pregnancy, drug–drug interactions, and environmental factors can notably influence the inter-individual variability of drug efficacy and adverse drug reactions^3,4^. In the past decades, the vast extent of genomic variation has been identified by whole-genome sequencing; the deciphering of genomic information has been conducted on an unprecedented scale worldwide, allowing the rapid progress of sequencing technologies^5^. Over 80% of all variations in pharmacogenes are newly discovered and observed at low frequencies across their respective populations; therefore, their impact on pharmacokinetics has not been evaluated^6^. Taken together, these rare variants account for a considerable percentage of the whole population; the determination of their effects on drug response and adverse effects would be essential for the improvement of personalised medicine.

In vitro assays, using a heterologous expression system can evaluate enzyme activity under highly reproducible conditions while removing patient risk involving highly invasive procedures as well as eliminating the risk of developing adverse drug reactions. Thus far, various expression systems, including *E. coli*, bacteria, yeast, baculoviruses, and mammalian cell lines have been utilised to characterise the functional differences of CYP variants caused by single nucleotide variations^7^. There have been discrepancies among these findings, in part due to the use of different expression systems. Moreover, it is well known that post-translational modification processes differ between species, which impedes accurate comparisons and assessment^8,9^. Although it would be beneficial to use a heterologous expression system in mammalian cells to reveal the enzymatic activities of CYP variants in humans, the low CYP levels in mammalian cells would hinder the establishment of an effective expression system.

Exogenous DNA delivery into animal cells is a widely used process in biological sciences for the expression of functional recombinant proteins. However, when this same strategy is applied with mammalian cells, costs significantly increase due to low expression levels^10^. Polyethylenimine (PEI), one of the most extensively used cationic polymers in vitro*,* can be used for successfully transfecting several mammalian cell lines, for instance, HEK293 and Chinese hamster ovary (CHO) cells^11^. Additionally, it has been reported that 293FT cells are highly suitable for CYP expression compared to COS-7, HepG2, and 293T cells^12^. Several studies have used the pcDNA3.4 TOPO vector, containing a full-length CMV promoter and other expression elements, as it allows for higher expression than other pcDNA-based expression constructs, making it suitable to achieve higher levels of CYP expression in 293FT cells^13,14^.

The protoporphyrin IX synthesis pathway plays an essential role in the synthesis of haem, a source of the CYP holoprotein form^15^. 5-Aminolevulinic acid (5-ALA) synthesis is the rate-limiting step in this process, and the addition of 5-ALA would increase CYP expression and holoprotein content^15^. Previous studies showed that 5-ALA and iron ions (ferrous and ferric) improved CYP expression levels when using *E. coli* and a human gastric cancer cell line^16,17^*.* Moreover, the synthesis route of endogenous haem was by far more effective than the external addition of haem^16^. Thus far, there are few studies utilising carbon monoxide (CO)-difference spectra measurements, which can determine the holoprotein content in microsomal proteins of recombinant CYP-expressing mammalian cells. Holoprotein expression levels evaluated by CO-difference spectra analysis are necessary to assess enzymatic activity, as holoproteins but not apoproteins are determinants of this activity. Consequently, to achieve high CYP levels in mammalian cells, the inclusion of 5-ALA and iron ions would be required.

Electron transport by the redox partner NADPH-cytochrome P450 oxidoreductase (CPR) plays a vital role in the exhibition of CYP catalytic activity^2,18,19^. CPR provides the essential first electron, whereas the second electron may come directly from CPR or via cytochrome b_5_ (CYB)^20,21^. So far, successful co-expression systems expressing nine CYP isoforms and CPR have been reported^22^. Their activities were increased, compared to the expression of CYP alone, by co-expression of CYP and CPR using several co-expression systems including baculoviruses, *Pichia pastoris*, *E. coli.*, and CHO cells^22–25^. Although there are no studies on the mechanism underlying the benefits of CYP and CPR plasmid co-expression in mammalian cells, the co-transfected expression system may be a compelling approach to understand the extent of functional changes caused by CYP gene polymorphisms. In addition to CPR, electron transfer via CYB also plays a role in the improvement of enzymatic activities of several CYP isoforms, including CYP2D6 and CYP3A4^26^. Co-expression systems could be used to screen for drugs metabolised by CYP isoforms, as improved production levels of metabolites catalysed by CYP would be advantageous in future studies. Therefore, the co-expression of CYP, CPR, and CYB should be considered to achieve optimal conditions for the evaluation of enzymatic activity in vitro.

Therefore, we developed an expression system optimised for the evaluation of CYP holoprotein activity using 293FT cells, which are known to yield high protein levels. To identify the most suitable conditions for CYP expression, we optimised the (1) transfection reagents and conditions, (2) concentrations of 5-ALA and Fe^2+^ supplementation, (3) timing of supplement addition and the incubation period with the supplements, and (4) plasmid ratios of CYP, CPR, and CYB for co-transfection of 293FT cells (Supplementary Fig. 1). Moreover, the enzymatic activities of the three CYP isoforms (CYP1A2, CYP2C9, and CYP3A4), which are highly expressed in the human liver, were analysed in vitro using each CYP probe substrate.

## Results

### Optimisation of 293FT cell transfection

**Figure 1:**
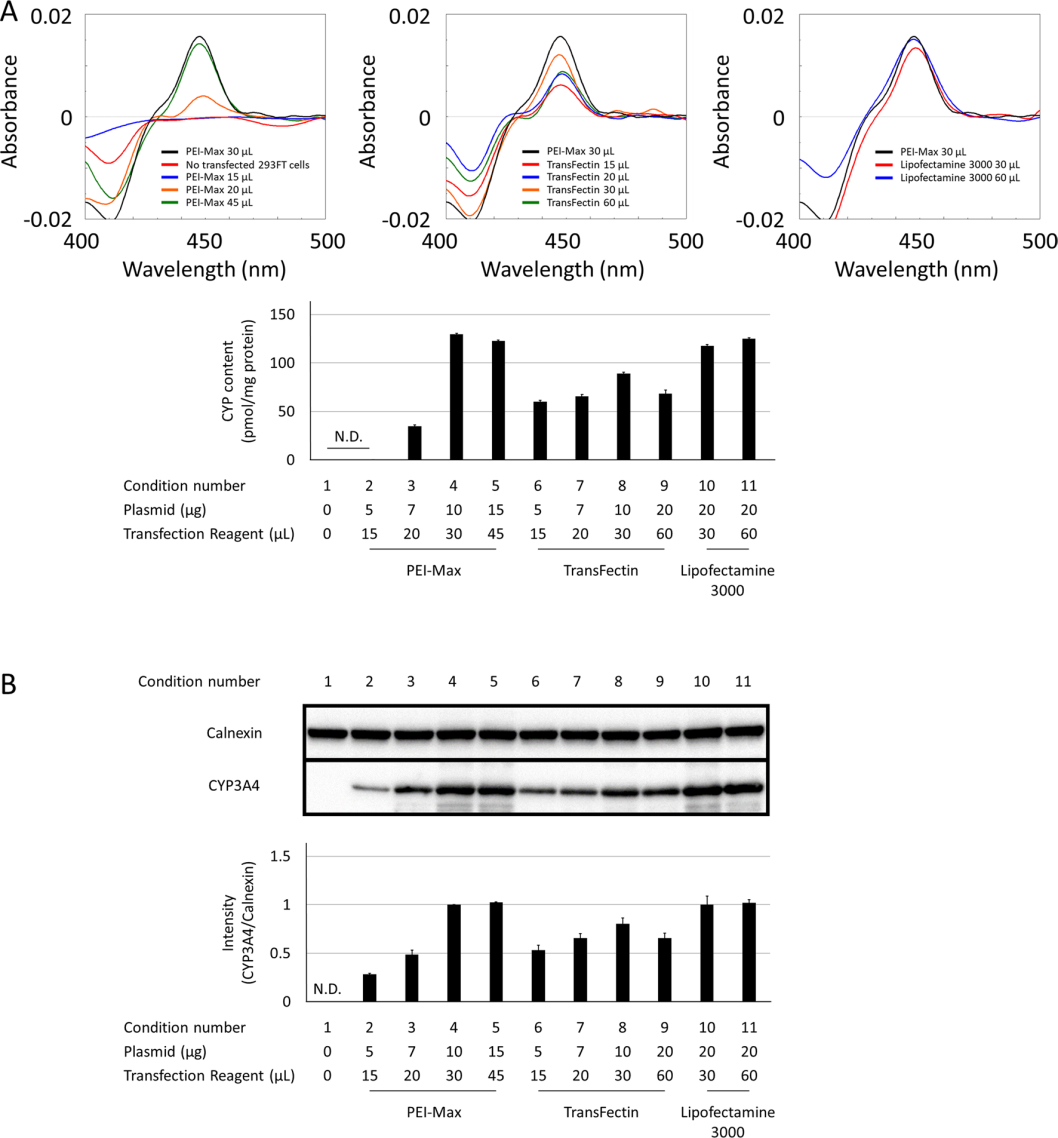
Transfection efficiency comparison of various transfection reagents (PEI-Max, Transfectin, and Lipofectamine 3000). 293FT cells were transfected with various DNA transfection reagent complexes described as condition numbers. The expression levels of CYP3A4 were determined at 48 h post-transfection by CO-difference spectroscopy (**A**) and western blotting, in which the PVDF membrane was cut across into two sections individually containing the protein of interest, CYP3A4 (57 kDa) and calnexin (90 kDa) for improved visualisation (**B**). *N.D.* not determined.

We first evaluated which transfection conditions were associated with the maximum CYP3A4 holoprotein expression levels in 293FT cells. CO-difference spectroscopy of microsomal fractions transfected with the CYP3A4 plasmid using different transfection reagents (PEI-Max, TransFectin, and Lipofectamine 3000) revealed that holoprotein levels were relatively higher with the utilisation of PEI-Max (30 and 45 μL) and Lipofectamine 3000 (Fig. 1A). Western blotting analysis verified that CYP3A4 proteins were detected under all transfection conditions (Fig. 1B) but were absent in non-transfected 293FT cells. Calnexin, an endoplasmic reticulum-resident protein, was used as a loading control^27^. Transfection cost-effectiveness was taken into consideration as a significant aspect of this study, and therefore, the 30 μL PEI-Max condition was selected.

### Additional optimisation of reagent concentrations

To improve CYP holoprotein expression in 293FT cells, we examined the impact of the cofactors 5-ALA and Fe^2+^ on CYP synthesis. The Soret peak at 450 nm was significantly increased by 0.05–1 mM 5-ALA or Fe^2+^ treatment for each measurement (Supplementary Figs. 2A and 2B). Separately, we examined the impact of combined addition for these cofactors. The combination of 0.25 mM 5-ALA and 0.25 mM Fe^2+^ showed the most substantial improvement of CYP3A4 holoprotein content compared to the use of each cofactor alone (Supplementary Fig. 2C). Although these treatments might also affect CYP3A4 total protein levels, as shown in the western blotting results (Supplementary Fig. 3), both 5-ALA and Fe^2+^ mainly play an influential role in CYP3A4 holoprotein synthesis more than impacting the regulation of CYP3A4 expression.

### Optimisation of post-transfection incubation times and effects of haem precursors on CYP expression

**Figure 2:**
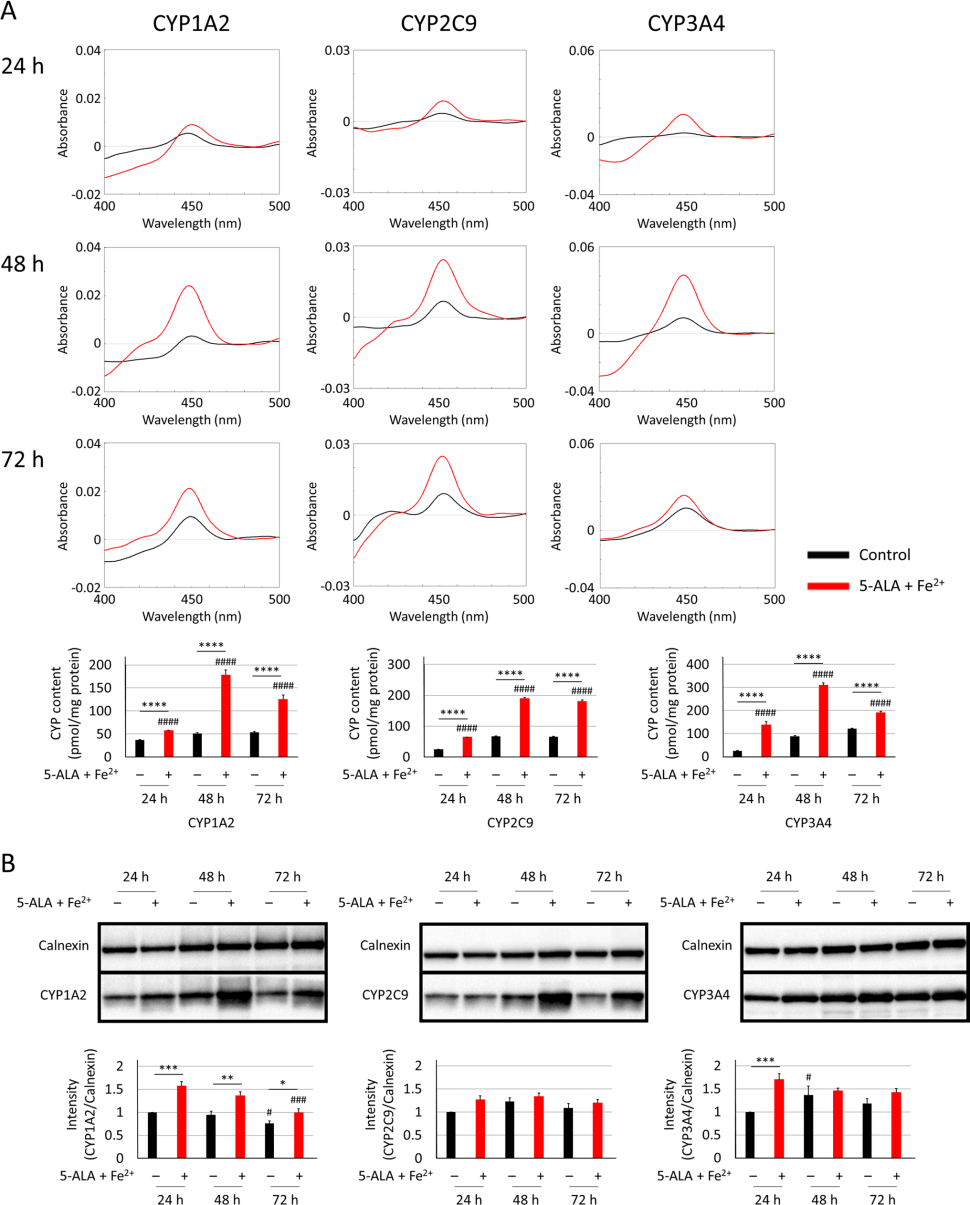
Optimisation of culture time following transfection. 293FT cells were transfected with 10 µg of each CYP plasmid (CYP1A2, CYP2C9, and CYP3A4) and 30 µL PEI-Max. The expression levels of CYP were determined at 24, 48, or 72 h post-transfection by CO-difference spectroscopy (**A**) and western blotting (**B**). PVDF membranes were cut into two sections individually containing CYP1A2 (58 kDa), CYP2C9 (56 kDa), or CYP3A4 (57 kDa) and calnexin (90 kDa). 5-ALA (0.25 mM) and Fe^2+^ (0.25 mM) were added or excluded at 12 h post-transfection. ^#^P < 0.05, ^###^P < 0.005, and ^####^P < 0.001 compared to 24 h group by Dunnett’s test. *P < 0.05, **P < 0.01, ***P < 0.005, and ****P < 0.001 compared to the additive-free control group by Dunnett’s test.

**Figure 3:**
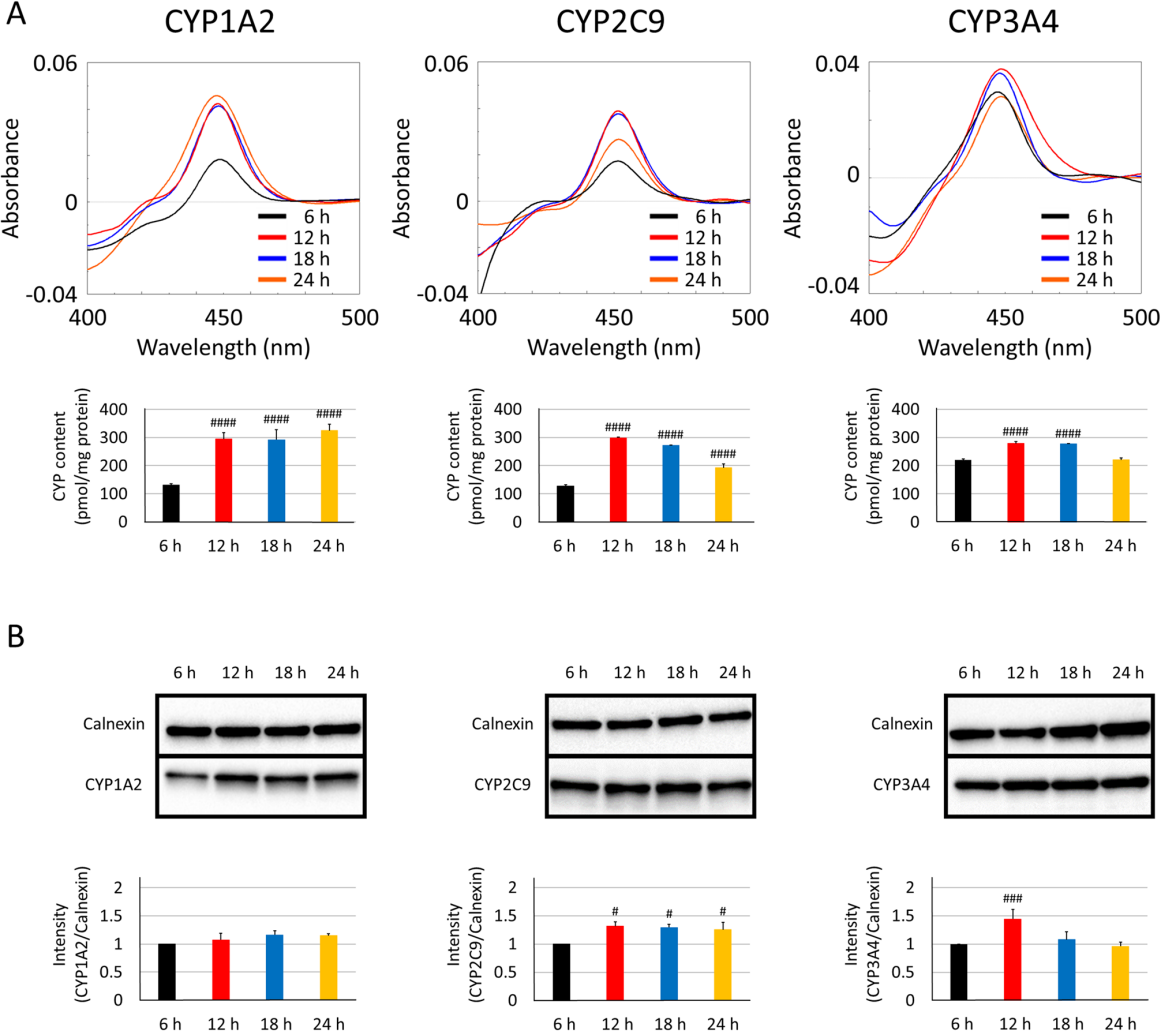
Optimisation of 5-ALA and Fe^2+^ supplementation. 293FT cells were transfected with 10 µg of each CYP plasmid (CYP1A2, CYP2C9, and CYP3A4) and 30 µL PEI-Max. The expression levels of CYP were determined at 48 h post-transfection by CO-difference spectroscopy (**A**) and western blotting (**B**). PVDF membranes were cut across into two sections individually containing CYP1A2 (58 kDa), CYP2C9 (56 kDa), or CYP3A4 (57 kDa) and calnexin (90 kDa). 5-ALA (0.25 mM) and Fe^2+^ (0.25 mM) were added at each post-transfection time point. ^#^P < 0.05, ^###^P < 0.005, and ^####^P < 0.001 compared to the 6 h group by Dunnett’s test.

First, the post-transfection incubation times for the three CYP isoforms (CYP1A2, CYP2C9, and CYP3A4) were evaluated by CO-difference spectra and western blotting. Figure 2A,B show the CO-difference spectra and western blot results for each CYP-containing microsomal fraction after 24, 48, and 72 h post-transfection incubation. A 48 h incubation period was necessary to obtain saturated CYP expression levels as observed by the CO-difference spectra for each CYP isoform. Furthermore, CYP holoprotein expression increased by the addition of 5-ALA and Fe^2+^ at each time point in these CYP isoforms. Next, we examined the impact of timing of both 5-ALA and Fe^2+^ treatments post-transfection; addition at 12 h post-transfection showed the highest holoprotein levels among 6, 12, 18, and 24 h post-transfection treatments (Fig. 3A,B).

### Optimisation of CYP and CPR and CYB plasmid co-transfection ratios in 293FT cells

**Figure 4:**
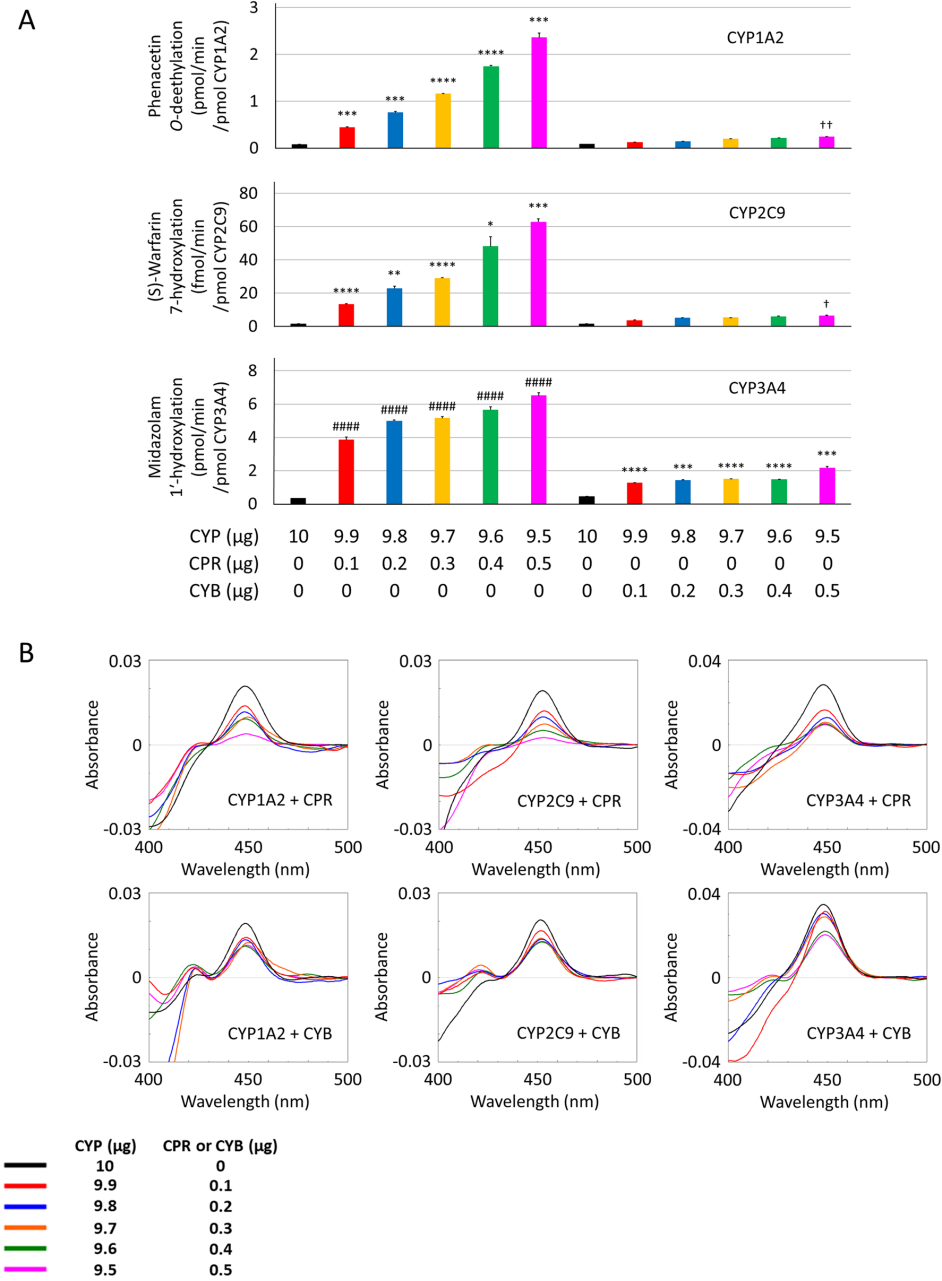
Optimisation of plasmid ratio for co-transfection. 293FT cells were transfected with each CYP plasmid (CYP1A2, CYP2C9, and CYP3A4) and CPR or CYB plasmid (total 10 μg) using 30 µL PEI-Max. Individual enzymatic activities of CYP1A2, CYP2C9, and CYP3A4 were quantified by LC–MS/MS or HPLC-fluorescence using phenacetin, (S)-warfarin, and midazolam (**A**). The expression levels of CYP were determined by CO-difference spectroscopy (**B**). ^####^P < 0.001 compared to 10 μg of each CYP plasmid transfected group by Dunnett's test. *P < 0.05, **P < 0.01, ***P < 0.005, and ****P < 0.001 compared to 10 μg of each CYP plasmid transfected group by Dunnett’s T3 test. ^†^P < 0.05 and ^††^P < 0.01 compared to 10 μg of each CYP plasmid transfected group by Kruskal–Wallis posthoc test.

CYP holoprotein levels improved in 293FT cells transfected with each CYP plasmid mentioned above. However, CPR and CYB electron transfer efficacy should be considered due to possible CYP holoprotein inefficacy as a result of insufficient electron transfer to CYP enzymes. Therefore, we investigated the influence of CYP co-transfection with both CPR and CYB using several plasmid ratios. As shown in Fig. 4A, the enzymatic activities for each CYP isoform were increased by co-transfection with the CPR plasmid.

Interestingly, the co-expression of CYP3A4 and CYB markedly increased CYP3A4-mediated midazolam 1′-hydroxylation. However, the CYP holoprotein levels decreased correspondingly with CYP plasmid amounts (Fig. 4B, Supplementary Fig. 4). CPR activities in the microsomal proteins coexpressed with both CYP and CPR correlated with the CPR plasmid amounts transfected into 293FT cells (Supplementary Fig. 5). Further, CYB content in the microsomal proteins coexpressed with both CYP and CYB correlated with the amount of CYB plasmid used for transfection (Supplementary Fig. 6). Western blotting analysis revealed that the expression of CYP, CPR, and CYB tended to correlate with the plasmid amounts (Supplementary Fig. 7). Although the turn-over was increased by CPR and CYB, CYP3A4 metabolite production was saturated by using 0.2 μg CPR plasmid alone, resulting in an overestimation of activity levels. Given the decreasing CO-difference spectra as a result of co-expression and the possible involvement of CYB in the improvement of electron transfer through CPR, the plasmid ratios of 9.8 (CYP) to 0.2 (CPR) and 9.8 (CYP) to 0.2 (CYB) were selected as compound settings for further evaluation of expression conditions.

**Figure 5:**
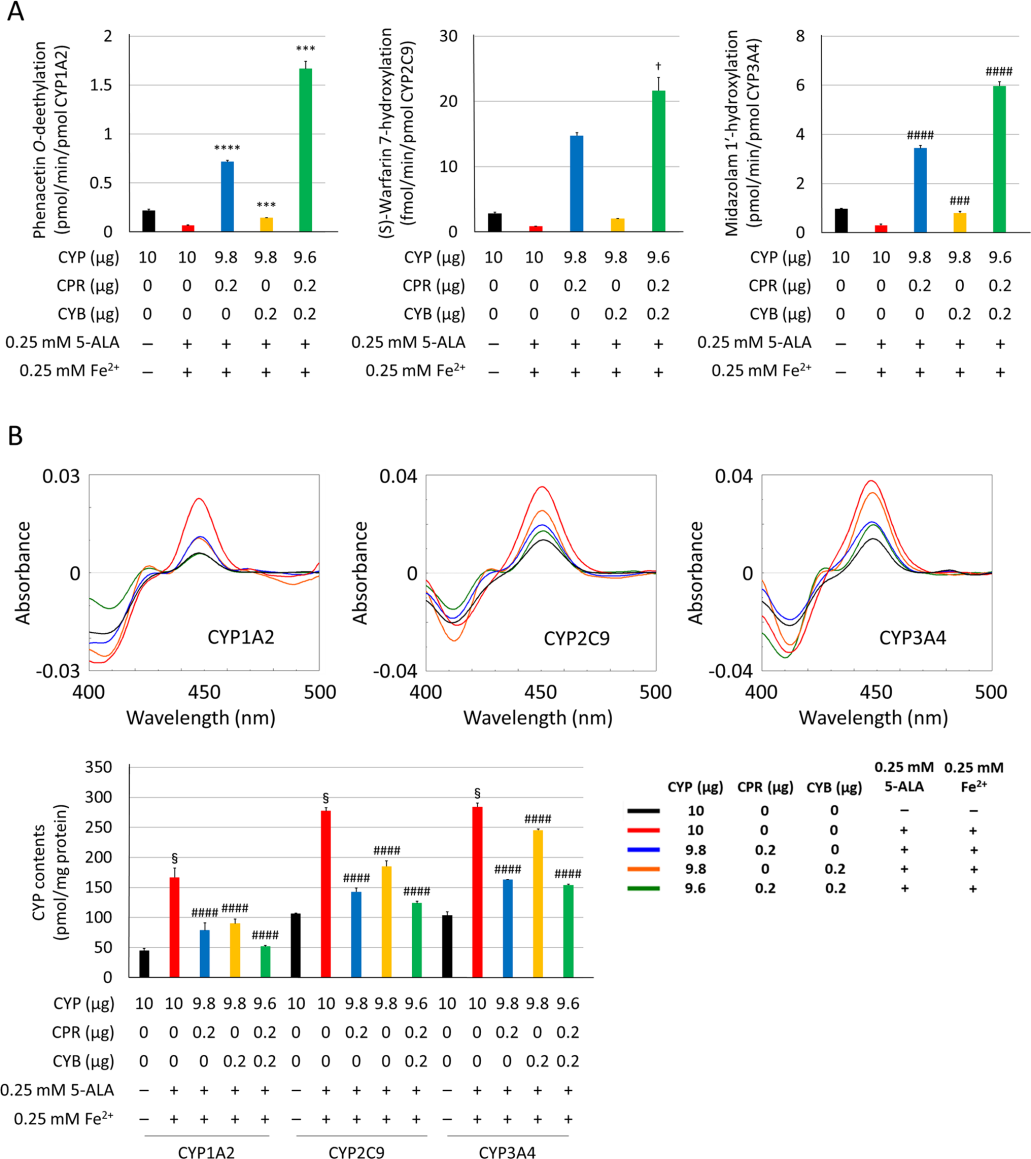
The overall impact of our cost-effective CYP expression optimisation method. Individual enzymatic activities of CYP1A2, CYP2C9, and CYP3A4 were quantified by LC–MS/MS or HPLC-fluorescence using phenacetin, (S)-warfarin, and midazolam (**A**). CYP expression levels were determined by CO-difference spectroscopy (**B**). ^§^P < 0.001 compared to the additive-free control group (black) by a two-tailed paired Student's t-test. ^###^P < 0.005 and ^####^P < 0.001 compared to 10 μg of each CYP plasmid transfected group (red) by Dunnett’s test. ***P < 0.005 and ****P < 0.001 compared to 10 μg of each CYP plasmid transfected group (red) by Dunnett’s T3 test. ^†^P < 0.05 compared to 10 μg of each CYP plasmid transfected group (red) by the Kruskal–Wallis posthoc test.

Finally, we confirmed the importance of the supplemental reagents (5-ALA and Fe^2+^) for co-transfection. Although the cofactors increased the holoprotein content in microsomal fractions, the enzymatic activities did not show a significant increase, which suggests that electron transfer to the CYP enzyme was insufficient with CYP overexpression alone (Fig. 5A,B). Notably, the enzymatic activities of microsomal proteins coexpressed with CYP, CPR, and CYB were enhanced to higher levels. CPR activities and CYB content quantified in the microsomal proteins were correlated with the CPR and CYB plasmid amounts transfected into 293FT cells, albeit the differences between the CYP isoforms (Supplementary Figs. 8 and 9). Western blotting analysis further revealed that the expression of CYP, CPR, and CYB tended to correlate with plasmid amounts (Supplementary Fig. 10).

## Discussion

CYPs as representative phase I enzymes are crucial for the prediction of inter-individual pharmacokinetics^2,3^. Amongst the several cDNA expression systems that have been successfully used for the functional characterisation of CYP allelic variants, mammalian cell expression systems would be preferable since the post-translational processing of proteins may influence enzymatic activity in some CYPs^7–9^. However, they have the disadvantage of producing low CYP protein levels. As a result, evaluating the metabolism of low concentration compounds and the measurement of their CO-difference spectra is impeded. In this study, we optimised the heterologous expression system for assessment of CYP enzymatic activity under high-precision conditions, using mammalian cells co-transfected with CPR and CYB plasmids.

First, we explored various transfection reagents and conditions to obtain the highest expression of CYP3A4 while attempting to maintain a low-cost heterologous expression system. Several studies have used Lipofectamine 3000 as a transfection reagent for various cell lines, whereas in our previous studies, TransFectin was used as a suitable transfection reagent, and an economical and efficient substitute for Lipofectamine 3000^14,28^. Recently, PEI-Max has been well acknowledged as a cost-effective transfection reagent successfully used on several kinds of mammalian cell lines such as HEK293 and CHO cells ^11,29^. In the present study, CYP3A4 amounts in 293FT cells transfected using PEI-Max showed similar levels compared to those obtained by utilising Lipofectamine 3000. For instance, PEI-Max costs less than 0.1 USD per 100-mm dish, whereas TransFectin and Lipofectamine 3000 costs 10 USD and 45 USD, respectively. Our study further supports PEI-Max as a cost-effective transfection reagent in comparison to others of the same category using mammalian cell lines.

5-ALA and Fe^2+^ are essential factors in the constitution and activation mechanisms of CYPs^15–17^. Notably, the addition of 5-ALA substantially improved holoprotein formation, as the 5-ALA synthesis process is recognised as the rate-limiting step to this formation. In addition to 5-ALA, supplementation with Fe^2+^ also contributed synergistically to the improvement, and thus the combinational addition of 5-ALA and Fe^2+^ considerably increased the holoenzyme content, more so than their separate individual addition. Next, we considered the post-transfection incubation time as well as the best timing for supplement addition that would allow for the purification of the highest holoprotein content, while taking into consideration the response lag in the case of transient CYP expression. The importance of 5-ALA and Fe^2+^ addition was ascertained by measuring the CO-difference spectroscopy after each post-transfection incubation time tested. We successfully determined that the addition of these supplements at 12 h post-transfection followed by a 48 h post-transfection cell incubation period is the optimal condition for CYP expression.

Electron transport to CYP by CPR is essential for CYPs to exhibit their catalytic activity^2,18,19^. The enzymatic activities of several CYP isoforms such as CYP2C8 and CYP3A4 are affected by the presence or absence of CYB^21,26^. As expected, the expression levels of CYP, CPR, and CYB were dependent on the transfected plasmid amounts. Notably, CYP expression levels were significantly decreased by CPR plasmid co-transfection, suggesting a CPR plasmid volume dependence. Reactive oxygen species (ROS) were generated from NADPH via the microsomal CYP-related electron transport system^30,31^. Increased ROS production by the overexpression of CPR could affect the stability of the CYP holoprotein, as indicated by the CO-difference spectra results for the co-expression of CYP and CPR (Fig. 4A). Interestingly, CPR expression and activity differed among coexpressed CYP isoforms, especially CYP1A2, suggesting that CPR saturating amounts differ between CYP isoforms. On the other hand, electron transfer via CYB appears to have played a partial role in CYP3A4 activity, observed as an increase in midazolam 1′-hydroxylation alongside CYB plasmid amounts. While CYP1A2 and CYP2C9, show a less significant contribution of CYB to electron transfer, suggesting that NADPH-dependent electron transfer directly via CPR may represent their enzymatic activities. Finally, combinational transfection caused an improvement in the enzymatic activities of all three CYP isoforms compared to transfection procedures using CPR and CYB alone. Our focus on enzymatic activity was due to the complex membrane anchor protein synthesis and regulation system, as well as the differences in culture conditions caused by plasmid transfection and cofactor supplementary addition. Importantly, there is no defined optimal heterologous expression system platform for pharmacokinetic assessment in humans to date. Mostly because of various observed discrepancies found across different research groups, including differences between in vitro and in vivo CYP regulation.

**Figure 6:**
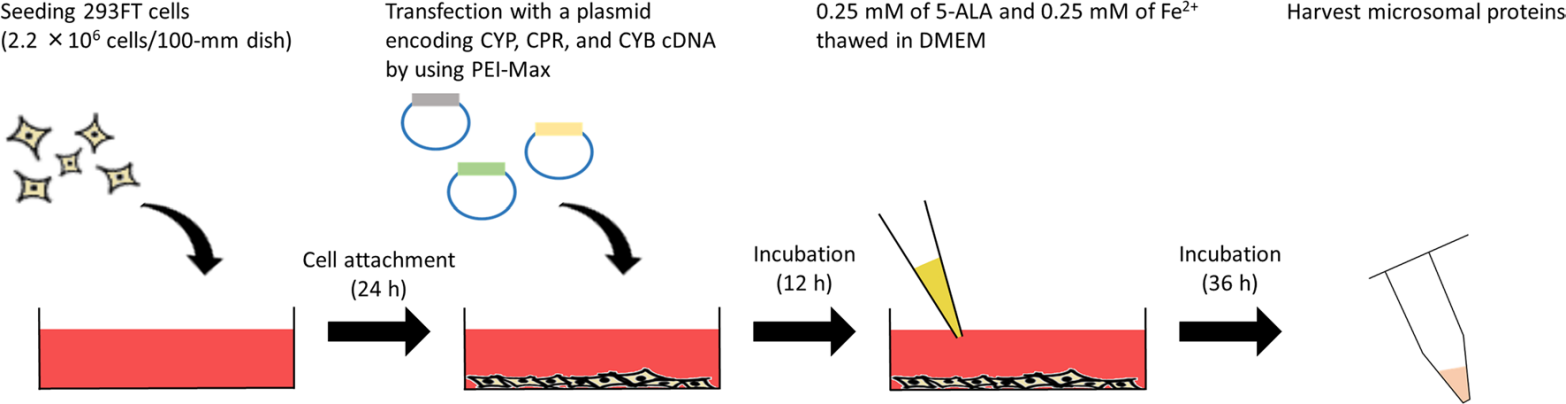
Optimised protocol for high-activity CYP expression established in this study.

In summary, we have established a beneficial heterologous expression system using 293FT cells co-transfected with CYP, CPR, and CYB plasmids (Fig. 6). This system achieved: (1) cost-effective CYP expression by using PEI-Max, (2) higher CYP expression levels, which can be accurately quantified by CO-difference spectroscopy, and (3) higher enzymatic activities compared to those obtained by overexpressing CYP alone. This heterologous expression system could be applied to evaluate the pharmacokinetics of clinically used and developing drugs. Moreover, the analysis of novel CYP low-frequency variants and variants previously known for exhibiting markedly decreased enzymatic activity could be improved, and its accuracy increased. However, this study encompassed only three CYP isoforms (CYP1A2, CYP2C9, and CYP3A4). Further investigation of additional clinically relevant isoforms such as CYP2C19 and CYP2D6 is necessary to validate this system further and support its widespread application. Furthermore, the kinetic parameters obtained by the enzymatic assays in vitro could be extended to pharmacokinetic parameters in vivo by linking to drug and metabolite plasma concentrations in patients, and thus this method could contribute to the further development and progression of personalised medicine, considering rare pharmacogenomic variability carriers.

## Methods

### Chemicals

The following reagents were purchased from the listed sources: phenacetin (Nacalai Tesque, Kyoto, Japan); 4-acetamidophenol, (S)-warfarin, and 7-ethoxycoumarin (Sigma-Aldrich, MO, USA); (S)-7-hydroxywarfarin (BD Gentest, Woburn, MA, USA); midazolam and flunitrazepam (Wako Pure Chemical Industries, Osaka, Japan); 1′-hydroxymidazolam (Corning Incorporated, Steuben County, NY, USA); 4-acetoamidophenol-d4 (Cayman Chemical, Ann Arbor, MI, USA); oxidised β-nicotinamide-adenine dinucleotide phosphate oxidised form (NADP^+^), glucose-6-phosphate (G-6-P), glucose-6-phosphate dehydrogenase (G-6-PDH), β-nicotinamide-adenine dinucleotide reduced form (NADH), and β-nicotinamide-adenine dinucleotide phosphate reduced form (NADPH) (Oriental Yeast, Tokyo, Japan); polyclonal anti-human CYP1A2 antibody, polyclonal anti-human CYP2C9 antibody, and polyclonal anti-human CYP3A4 antibody (Nosan Corporation); polyclonal anti-calnexin antibody (Enzo Life Sciences); anti-cytochrome P450 reductase antibody (ab13513) and anti-cytochrome b_5_ antibody (ab69801) (Abcam, CB, UK); horseradish peroxidase (HRP)-conjugated goat anti-rabbit IgG (DakoCytomation, Glostrup, Denmark); dimanganese decacarbonyl (DMDC) (Sigma-Aldrich, Steinheim, Germany); sodium cyanide and cytochrome c from horse heart (Nacalai Tesque, Kyoto, Japan). All other chemicals and reagents were of the highest commercially available quality.

### Construction of expression vectors

*CYP3A4* complementary DNA (cDNA) subcloned into the pENTR/D-TOPO vector was gifted by Prof. Mizuguchi's laboratory (Osaka University)^32^. *CYP1A2* and *CYP2C9* cDNAs subcloned into the pENTR/D-TOPO vector were prepared as previously described^33,34^. *CPR* cDNA fragments obtained from a human lung cDNA library (Takara, Shiga, Japan) were amplified by PCR with a forward primer (5′-CACCATGATCAACATGGGAGACTC-3′) and a reverse primer (5′-CTAGCTCCACACGTCCAGGGAGTG-3′) using PfuUltra High-Fidelity DNA polymerase (Agilent Technologies, Santa Clara, CA, USA). *CYB* cDNA fragments obtained from a human lung cDNA library (Takara) were amplified by PCR with a forward primer (5′-CACCATGGCAGAGCAGTCGGAC-3′) and a reverse primer (5′-TCAGTCCTCTGCCATGTATAGGCG-3′) utilising KAPA HiFi HotStart ReadyMix (KAPA Biosystems, MA, USA). The underlined sequences in the forward primer were introduced for directional TOPO cloning. Following amplification, the wild-type *CPR* and *CYB* fragments were subcloned into the pENTR/D-TOPO vector (ThermoFisher Scientific, Waltham, MA). All *CYP*, *CPR*, and *CYB* cDNAs were confirmed by Sanger sequencing. Each cDNA was subcloned into the mammalian expression vector pcDNA3.4 (ThermoFisher Scientific).

### CYP expression in 293FT cells

293FT cells purchased from ThermoFisher Scientific were cultured in Dulbecco's modified Eagle's medium (Nacalai Tesque) containing 10% foetal bovine serum at 37 °C under 5% CO_2_. Cells were plated at a density of 2.2 × 10^6^ cells/100-mm dish; 24 h after plating, cells were transfected with a plasmid encoding each *CYP*, *CPR*, and *CYB* cDNA using the TransFectin lipid reagent (Bio-Rad Laboratories, Hercules, CA, USA). Lipofectamine 3000, or 1.0 mg/mL polyethyleneimine "Max" (PEI-Max) (Polysciences, Inc., Warrington, PA, USA) according to the manufacturer's instructions. The 293FT cells were scraped off, and microsomal fractions were prepared as previously described^28^. Collected cells were centrifuged at 1,500 × *g* for 5 min and resuspended in 10 mM Tris–HCl (pH 7.4) containing 1 mM EDTA and 10% glycerol. Microsomal fractions were obtained by differential centrifugation at 9,000 × *g* for 20 min, the resulting supernatant was subsequently centrifuged at 105,000 × *g* for 60 min. The microsomal pellet was resuspended in 50 mM Tris–HCl (pH 7.4) containing 1 mM EDTA, 20% glycerol, 150 mM KCl, and Protease Inhibitor Cocktail for Use with Mammalian Cell and Tissue Extract (NacalaiTesque).

### Microsomal CYP content determination

Protein concentrations were determined using a BCA protein assay kit (ThermoFisher Scientific). The CYP holoprotein content was measured spectrophotometrically according to previously reported methods with several modifications^35,36^. The microsomal fraction (0.3 mg) was diluted to 200 μL in 100 mM potassium phosphate buffer (pH 7.4) containing 20% glycerol (v/v). Ninety-nine microliters of sample protein were added to the sample and reference cuvettes. A baseline between 400 and 500 nm was recorded using a Cary 300 UV–Vis spectrophotometer (Agilent Technologies). The sample cuvette was illuminated with over 20,000 lx LED-Light for 30 s after the addition of 1 μL 20 mM DMDC diluted in DMSO. Samples containing dithionite were reduced by using approximately 0.2 mg solid Na_2_S_2_O_4_ for both cuvettes. The absorbance spectra of both cuvettes were then spectrophotometrically recorded between 400 and 500 nm. All assays and measurements were performed in triplicate using a single microsomal preparation. Data analysis was conducted using Jasco Spectra Manager (JASCO Corporation, Sendai, Japan). The CYP concentration was calculated utilising the maximum absorbance of three separate records employing the extinction coefficient of the CO-difference spectrum (91 mM^−1^ per cm at 450 nm). Cuvettes (Sub-Micro Cells, 16.50-Q-10/Z20) were purchased from Starna Scientific, Ltd. (London, UK).

### Western blotting

Five micrograms of 293FT microsomal fractions were separated by 10% sodium dodecyl sulfate–polyacrylamide gel electrophoresis (SDS-PAGE). Western blotting was performed in triplicates using a single microsomal preparation according to standard procedures. CYP, CPR, CYB, and calnexin were detected using the antibodies described in Supplementary Table 1. Detection was achieved utilising a secondary HRP-conjugated goat anti-rabbit IgG (1:10,000). Immunoblots were visualised using SuperSignal West Pico PLUS Chemiluminescent Substrate (ThermoFisher Scientific) for each CYP (CYP1A2, CYP2C9, and CYP3A4) and SuperSignal West Femto Maximum Sensitivity Substrate (ThermoFisher Scientific) for CPR, CYB, and calnexin, respectively. Chemiluminescence was quantified using a ChemiDoc XRS + system with Image Lab Software (Bio-Rad Laboratories).

### Measurement of NADPH-cytochrome c reduction activity

CPR activity was evaluated according to previously described methods with minor modifications^36,37^. The microsomal fraction (1 μg microsomal protein) and 400 μM cytochrome c were diluted to 180 μL in 100 mM potassium phosphate buffer (pH 7.4) containing 1.0 mM sodium cyanide. Protein samples (90 μL each) were added to the sample and reference cuvettes. A blank was recorded at 550 nm using a Cary 300 UV–Vis spectrophotometer (Agilent Technologies). Reactions were initiated by the addition of 1.0 mM NADPH diluted in 100 mM potassium phosphate buffer (pH 7.4) containing 1.0 mM sodium cyanide. The control sample contained all reagents except NADPH in a reference cuvette. The absorbance for the microsomal fractions expressing each CYP and CPR was recorded for 2.5 min. The activity of CPR toward cytochrome c was calculated using the extinction coefficient of cytochrome c (21 mM^−1^ per cm at 550 nm). All experiments were performed in triplicates using a single microsomal preparation.

### Microsomal CYB content determination

CYB content was measured spectrophotometrically according to previously reported methods with several modifications^35^. The microsomal fraction (50 μg) was diluted to 200 μL in 100 mM potassium phosphate buffer (pH 7.4) containing 1.0 mM sodium cyanide. Ninety microliters of sample protein were added to the sample and reference cuvettes. A baseline between 400 and 500 nm was recorded using a Cary 300 UV–Vis spectrophotometer (Agilent Technologies). After the addition of 10 μL 4 mM NADH, the absorbance spectra of both cuvettes were spectrophotometrically recorded between 400 and 500 nm. All assays and measurements were performed in triplicates using a single microsomal preparation. Data analysis was conducted using a Jasco Spectra Manager (JASCO Corporation, Sendai, Japan). CYB concentrations were calculated utilising the absorbance of three separate recordings employing reduced minus oxidised difference spectra at 424 nm (185 mM^−1^ per cm).

### Assays for the catalytic activity of CYP isoforms

All catalytic assays were performed in triplicates using a single microsomal preparation.

### Phenacetin *O*-deethylation

CYP1A2-mediated phenacetin *O*-deethylation was evaluated as previously reported with several modifications^34^. The reaction mixture (150 µL) contained the microsomal fraction (50 µg), 500 µM phenacetin, 3.3 mM MgCl_2_, and 50 mM potassium phosphate buffer (pH 7.4). Following pre-incubation at 37 °C for 3 min, reactions were initiated by the addition of an NADPH-generating medium consisting of 1.3 mM NADP^+^, 3.3 mM G-6-P, and 0.4 U/mL G-6-PDH. After incubation at 37 °C for 40 min, reactions were terminated by the addition of 150 μL acetonitrile containing 5 μM acetaminophen-d4 (internal standard). Protein was removed from the reaction tube by centrifugation at 15,400 × *g* for 10 min. Five microliters of the supernatant were injected into a liquid chromatography-tandem mass spectrometry (LC–MS/MS) system.

Chromatographic separation was achieved using a Luna C18 column (2.0 × 150 mm, 5-μm particle size) maintained at 40 °C. Mobile phases consisted of deionised water containing 0.1% formic acid as eluent A, and acetonitrile containing 0.1% formic acid as eluent B, passed through a NANOSPACE SI-2 HPLC System (OSAKA SODA, Osaka, Japan) at a constant 300 μL/min flow rate for sample analysis. The gradient program was as follows: initial elution with 100% A, followed by a linear gradient to 90% B from 1 to 10 min, held at 100% B for 2 min, and then immediately returned to the initial conditions and maintained for 3 min. Acetaminophen was measured in positive ion detection mode at the electrospray ionisation interface (TSQ Quantum Ultra, Thermo Fisher Scientific) and quantified using the Xcalibur software (Thermo Fisher Scientific). Quantification analyses were performed in the selected reaction monitoring mode, in which ion transitions from the precursor into product ion were monitored, m/z 179.9 → 111.0 for phenacetin (collision energy, 21 V), m/z 151.9 → 110.0 for acetaminophen (collision energy, 16 V), and m/z 155.9 → 114.1 for acetaminophen-d4 (collision energy, 17 V). The optimised parameters for MS were as follows: positive HESI spray voltage, 3.0 kV; heated capillary temperature, 300 °C; sheath gas pressure, 50 Arb; auxiliary gas setting, 15 Arb; and a heated vaporiser temperature of 450 °C. Nitrogen was used as both the sheath and auxiliary gas and argon as the collision gas at 1.5 mTorr. Standard curves for acetaminophen were constructed in a 50–10,000 nM range using metabolite standards, with a quantification limit of 50 nM. The compound concentrations of all samples were within the linearity range.

### (S)-warfarin 7-hydroxylation

Warfarin 7-hydroxylation by CYP2C9 was measured as previously reported with several modifications^33^. The reaction mixture (150 μL) contained the microsomal fraction (25 μg), 40 μM (S)-warfarin, 3.3 mM MgCl_2_, and 100 mM potassium phosphate buffer (pH 7.4). Following pre-incubation at 37 °C for 3 min, reactions were initiated by the addition of 1 mM β-NADPH. After incubation at 37 °C for 60 min, reactions were terminated by the addition of 150 μL acetonitrile containing 12.5 nM 7-ethoxycoumarin (internal standard). After protein removal by centrifugation at 15,400 × *g* for 10 min, 50 μL supernatant was injected into an HPLC system consisting of a Waters 2,695 separations module, a Waters 2,475 multi λ fluorescence detector (Waters, Milford, MA, USA), and a SunFire C18 column (4.6 × 150 mm, 5-μm particle size; Waters) maintained at 40 °C. The mobile phase was a mixture of acetonitrile and 0.5% phosphoric acid (38:62, v/v) at a flow rate of 1.0 mL/min. (S)-7-hydroxywarfarin content was measured at an excitation wavelength of 320 nm and an emission wavelength of 415 nm. Standard curves for 7-hydroxycoumarin were constructed in the 0.5–64 nM range using authentic metabolite standards, and the lower limit of (S)-7-hydroxywarfarin quantification was 0.5 nM. The compound concentrations of all samples were within the linearity range.

### Midazolam 1′-hydroxylation

The midazolam 1′-hydroxylation by CYP3A4 was determined according to previously reported protocols with several modifications^38^. The reaction mixture (100 μL) contained the microsomal fraction (50 μg), 10 μM midazolam, 20 mM MgCl_2_, and 100 mM potassium phosphate buffer (pH 7.4). Following pre-incubation at 37 °C for 3 min, reactions were initiated by the addition of 10 mM β-NADPH. After incubation at 37 °C for 10 min, reactions were terminated by the addition of 100 μL acetonitrile containing 500 nM flunitrazepam (internal standard). Protein was removed by centrifuging the reaction tube at 15,400 × *g* for 10 min and 5 μL of the supernatant was injected into an LC–MS/MS system.

1′-Hydroxymidazolam was measured by the same LC–MS/MS system, mobile phases, and flow rate as specified for phenacetin *O*-deethylation. Chromatographic separation was performed using an XBridge C18 column (2.1 × 100 mm, 3.5-μm particle size; Waters) maintained at 40 °C. The gradient program was as follows: initial elution with 100% A, followed by a linear gradient to 90% B from 1 to 10 min, held at 100% B for 2 min, and then immediately returned to initial conditions and maintained for 3 min. Quantification analyses were performed in the selected reaction monitoring mode, in which ion transitions from the precursor into product ion were monitored: m/z 326.0 → 291.2 for midazolam (collision energy, 28 V), m/z 342.0 → 323.9 for 1′-hydroxymidazolam (collision energy, 21 V), and m/z 314.0 → 268.1 for flunitrazepam (collision energy, 26 V). The optimised parameters for MS were as follows: positive HESI spray voltage, 3.5 kV; heated capillary temperature, 300 °C; sheath gas pressure, 40 Arb; auxiliary gas setting, 15 Arb; and heated vaporiser temperature, 450 °C. Nitrogen was used as both the sheath and auxiliary gas and argon as the collision gas at 1.5 mTorr. The lower 1′-hydroxymidazolam quantification limit was 10 nM. Standard curves for 1′-hydroxymidazolam were constructed in the 50–10,000 nM range using authentic metabolite standards. The compound concentrations of all samples were within the linearity range.

### Statistics

A two-tailed paired Student's *t*-test was performed to compare data between the two groups. The normality of our datasets was initially assessed using the Shapiro–Wilk Test. Statistical analyses for multiple comparisons were performed through variance analysis by Dunnett's test*,* Dunnett's T3 test, or the Kruskal–Wallis method (IBM SPSS Statistics Ver. 22, International Business Machines, Armonk, NY, USA). All values are expressed as the mean ± SD of experiments performed in triplicate. Differences with P < 0.05 were considered statistically significant. All assays and samples were prepared in triplicates to permit statistical analysis.

## Supplementary information

Supplementary figure legends.

Supplementary figures.

Supplementary table 1.
