# Supplementary material for: Heterologous expression of high-activity cytochrome P450 in mammalian cells
Source: Sci Rep. 2020 Aug 25;10:14193. doi: 10.1038/s41598-020-71035-5 (PMC7447777; doi:10.1038/s41598-020-71035-5)
Supplement: Supplementary file 1 — Supplementary figure legends. [file 41598_2020_71035_MOESM1_ESM.docx]

Supplementary Figure 1. Overview of the considerations for high-activity CYP expression in this study.

Supplementary Figure 2. Optimisation of additional supplement concentrations and consideration of the combination of 5-ALA and Fe^2+^. 293FT cells were transfected with 10 µg CYP3A4 and 30 µL PEI-Max. CYP3A4 expression levels were determined at 48 h post-transfection by CO-difference spectroscopy. Each concentration of 5-ALA and Fe^2+^ was added at 12 h post-transfection. ^#^P < 0.05, ^##^P < 0.01, ^###^P < 0.005, and ^####^P < 0.001 compared to the additive-free control by Dunnett’s test. ^*^P < 0.05 compared to the additive-free control group by Kruskal-Wallis posthoc test.

Supplementary Figure 3. Optimisation of additional supplement concentrations and consideration of the combination of 5-ALA and Fe^2+^. 293FT cells were transfected with 10 µg CYP3A4 and 30 µL PEI-Max. CYP3A4 expression levels were determined at 48 h post-transfection by western blotting; PVDF membranes were cut into two sections individually containing CYP3A4 (57 kDa) and calnexin (90 kDa). Each concentration of 5-ALA and Fe^2+^ was added at 12 h post-transfection. ^#^P < 0.05 compared to the additive-free control by the Dunnett's test.

Supplementary Figure 4. Optimisation of plasmid ratio for co-transfection. 293FT cells were transfected with each CYP plasmid (CYP1A2, CYP2C9, and CYP3A4) and CPR or CYB plasmid (total 10 μg) using 30 µL PEI-Max. CYP contents were determined by CO-difference spectroscopy. ^#^P < 0.05, ^##^P < 0.01, ^###^P < 0.005, and ^####^P < 0.001 compared to 10 μg of each CYP plasmid transfected group by Dunnett’s test. ^*^P < 0.05, ^**^P < 0.01, and ^****^P < 0.001 compared to 10 μg of each CYP plasmid transfected group by Dunnett’s T3 test.

Supplementary Figure 5. Optimisation of plasmid ratio for co-transfection. 293FT cells were transfected with each CYP plasmid (CYP1A2, CYP2C9, and CYP3A4) and CPR plasmid (total 10 μg) using 30 µL PEI-Max. CPR activities were evaluated by cytochrome c reduction assay using 1.0 μg microsomal protein. ^#^P < 0.05, ^##^P < 0.01, and ^####^P < 0.001 compared to 10 μg of each CYP plasmid transfected group by Dunnett’s test. ^*^P < 0.05, ^**^P < 0.01, and ^****^P < 0.001 compared to 10 μg of each CYP plasmid transfected group by Dunnett’s T3 test.

Supplementary Figure 6. Optimisation of plasmid ratio for co-transfection. 293FT cells were transfected with each CYP plasmid (CYP1A2, CYP2C9, and CYP3A4) and CYB plasmid (total 10 μg) using 30 µL PEI-Max. CYB amounts in microsomal protein were measured by reduced minus oxidised difference spectroscopy. ^#^P < 0.05, ^###^P < 0.005, and ^####^P < 0.001 compared to 10 μg of each CYP plasmid transfected group by Dunnett’s test.

Supplementary Figure 7. Optimisation of plasmid ratio for co-transfection. 293FT cells were transfected with each CYP plasmid and CPR or CYB plasmid (total 10 μg) using 30 µL PEI-Max. Western blotting for microsomal proteins showed the expression levels of each CYP (56-58 kDa), CPR (70 kDa), CYB (16 kDa), and calnexin (90 kDa). PVDF membranes were cut across into three individual sections containing CYP, calnexin, and either CPR or CYB, respectively.

Supplementary Figure 8. The overall impact of our cost-effective CYP expression optimisation method. CPR activities were evaluated by cytochrome c reduction assay using 1.0 μg microsomal protein. ^###^P < 0.005 and ^####^P < 0.001 compared to 10 μg of each CYP plasmid transfected group (red) by Dunnett’s test.

Supplementary Figure 9. The overall impact of our cost-effective CYP expression optimisation method. CYB amounts in microsomal protein were measured by reduced minus oxidised difference spectroscopy. ^####^P < 0.001 compared to 10 μg of each CYP plasmid transfected group (red) by Dunnett’s test. ^*^P < 0.05, ^**^P < 0.01, and ^****^P < 0.001 compared to 10 μg of each CYP plasmid transfected group (red) by Dunnett’s T3 test.

Supplementary Figure 10. The overall impact of our cost-effective CYP expression optimisation method. Western blotting of microsomal proteins showed the expression levels of each CYP (56-58 kDa), CPR (70 kDa), CYB (16 kDa), and calnexin (90 kDa). PVDF membranes were cut across into three individual sections containing CYP, calnexin, and either CPR or CYB, respectively.
