# Supplementary material for: Heterologous expression of high-activity cytochrome P450 in mammalian cells
Source: Sci Rep. 2020 Aug 25;10:14193. doi: 10.1038/s41598-020-71035-5 (PMC7447777; doi:10.1038/s41598-020-71035-5)

Heterologous expression of high-activity cytochrome P450 in mammalian cells

Masaki Kumondai[1], Eiji Hishinuma[1, 2, 3], Evelyn Marie Gutiérrez Rico[1], Akio Ito[1], Yuya Nakanishi[1], Daisuke Saigusa[3], Noriyasu Hirasawa[1, 2, 4], Masahiro Hiratsuka[1, 2, 3, 4, *]

[1]Laboratory of Pharmacotherapy of Life-Style Related Diseases, Graduate School of Pharmaceutical Sciences, Tohoku University, 6-3 Aoba Aramaki, Aoba-ku, Sendai 980-8578, Japan

[2]Advanced Research Center for Innovations in Next-Generation Medicine, Tohoku University, 2-1 Seiryo-machi, Aoba-ku, Sendai 980-8575, Japan

[3]Tohoku Medical Megabank Organization, Tohoku University, 2-1 Seiryo-machi, Aoba-ku, Sendai 980-8573, Japan

[4]Department of Pharmaceutical Sciences, Tohoku University Hospital, 1-1 Seiryo-machi, Aoba-ku, Sendai 980-8574, Japan

*Corresponding author:

Dr. Masahiro Hiratsuka, Ph.D.

Laboratory of Pharmacotherapy of Life-Style Related Diseases, Graduate School of Pharmaceutical Sciences, Tohoku University, 6-3, Aoba, Aramaki, Aoba-ku, Sendai 980-8578, Japan

Tel & Fax: +81-22-717-7049

E-mail: masahiro.hiratsuka.a8@tohoku.ac.jp

Supplementary Figure 1

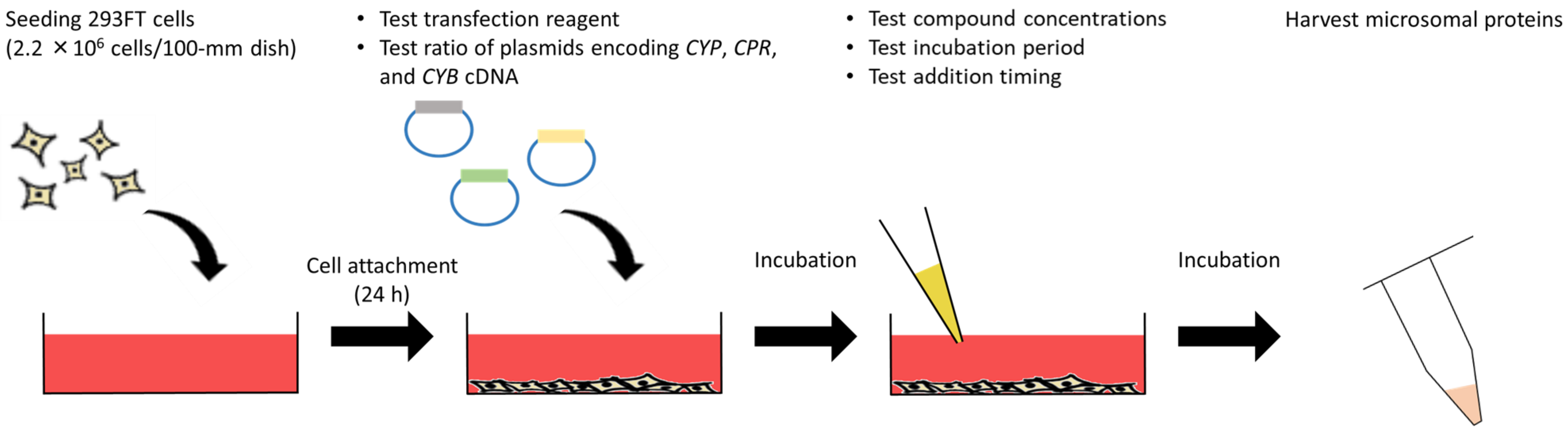

Supplementary Figure 2

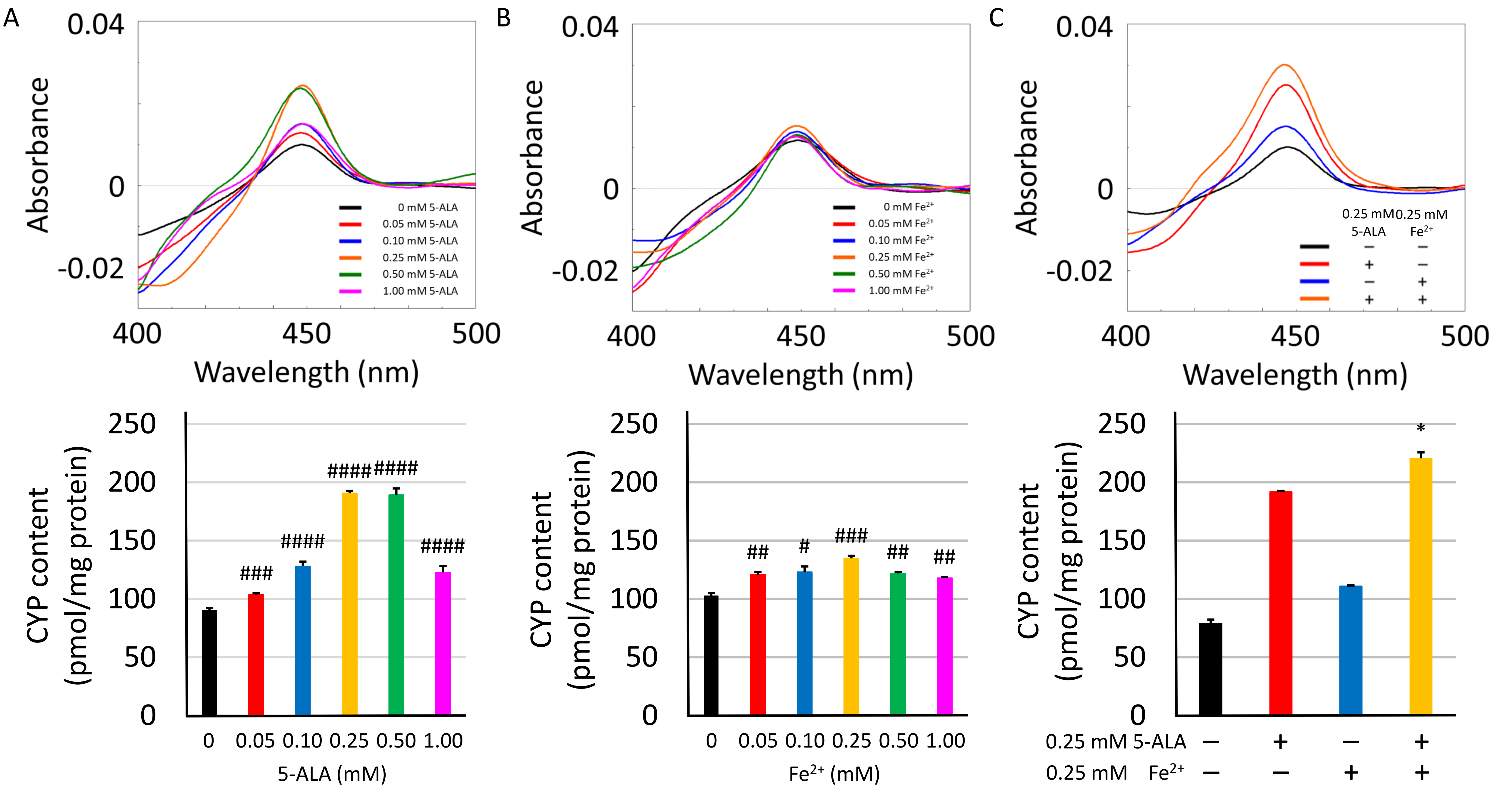

Supplementary Figure 3

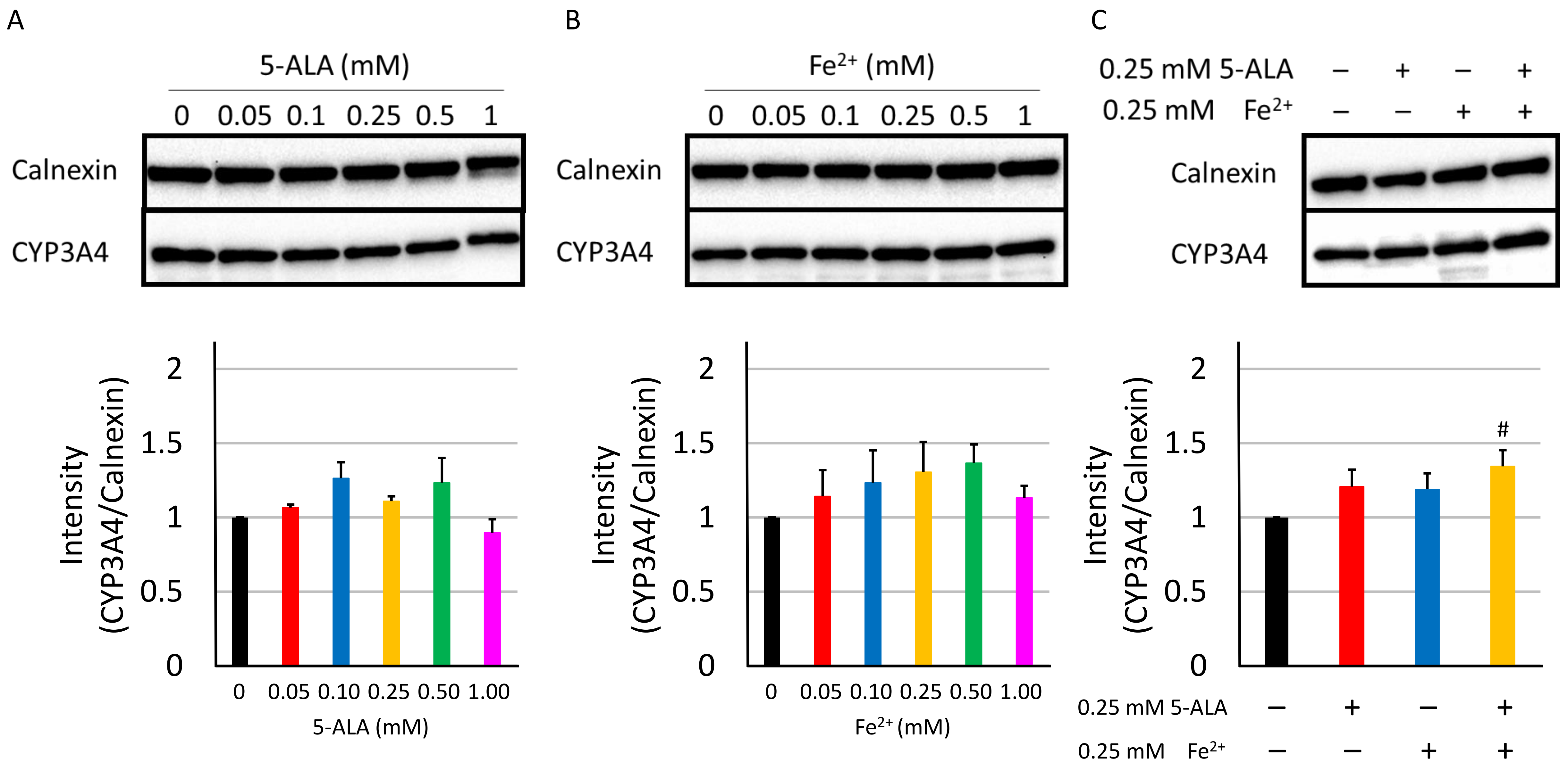

Supplementary Figure 4

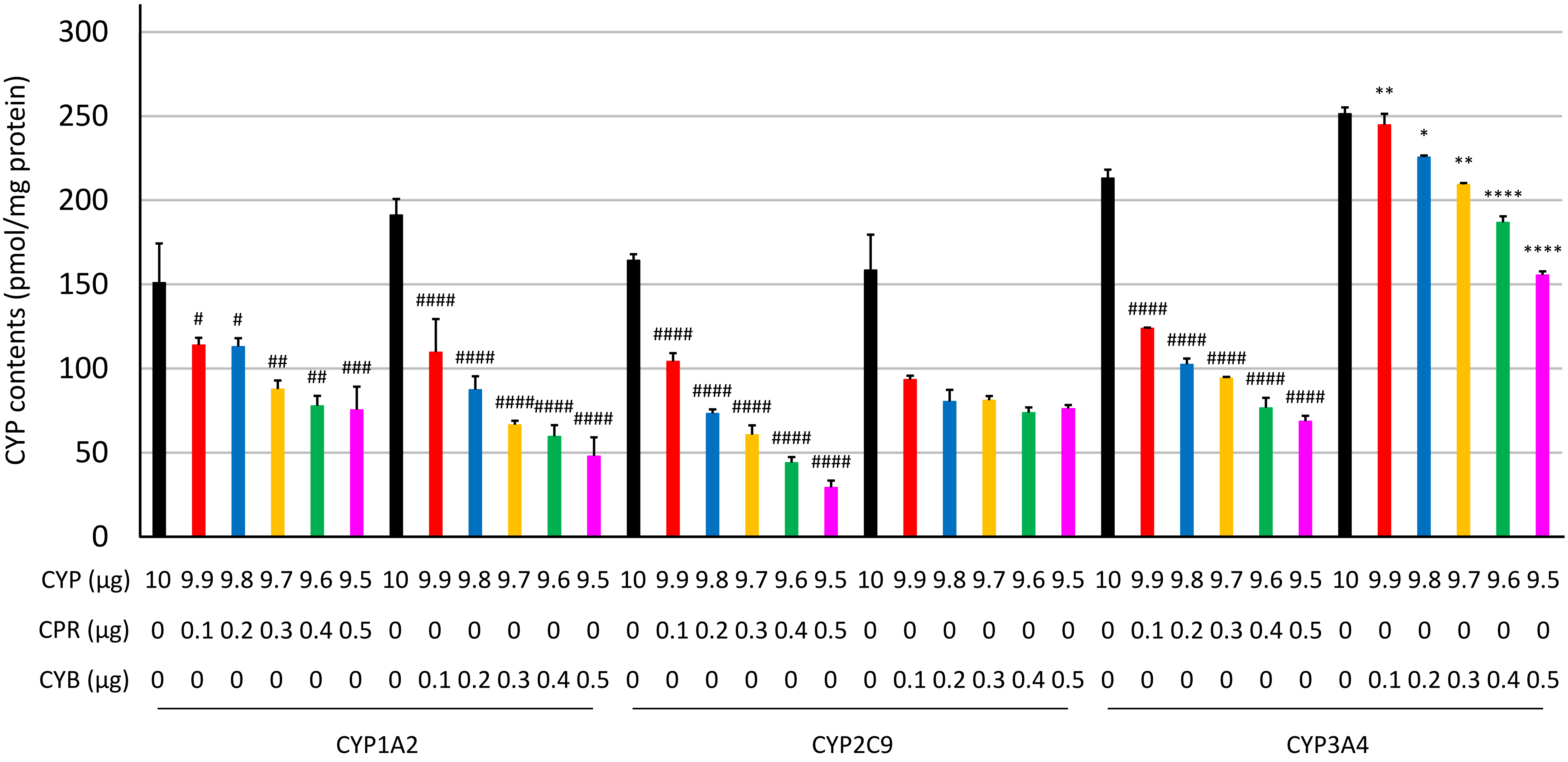

Supplementary Figure 5

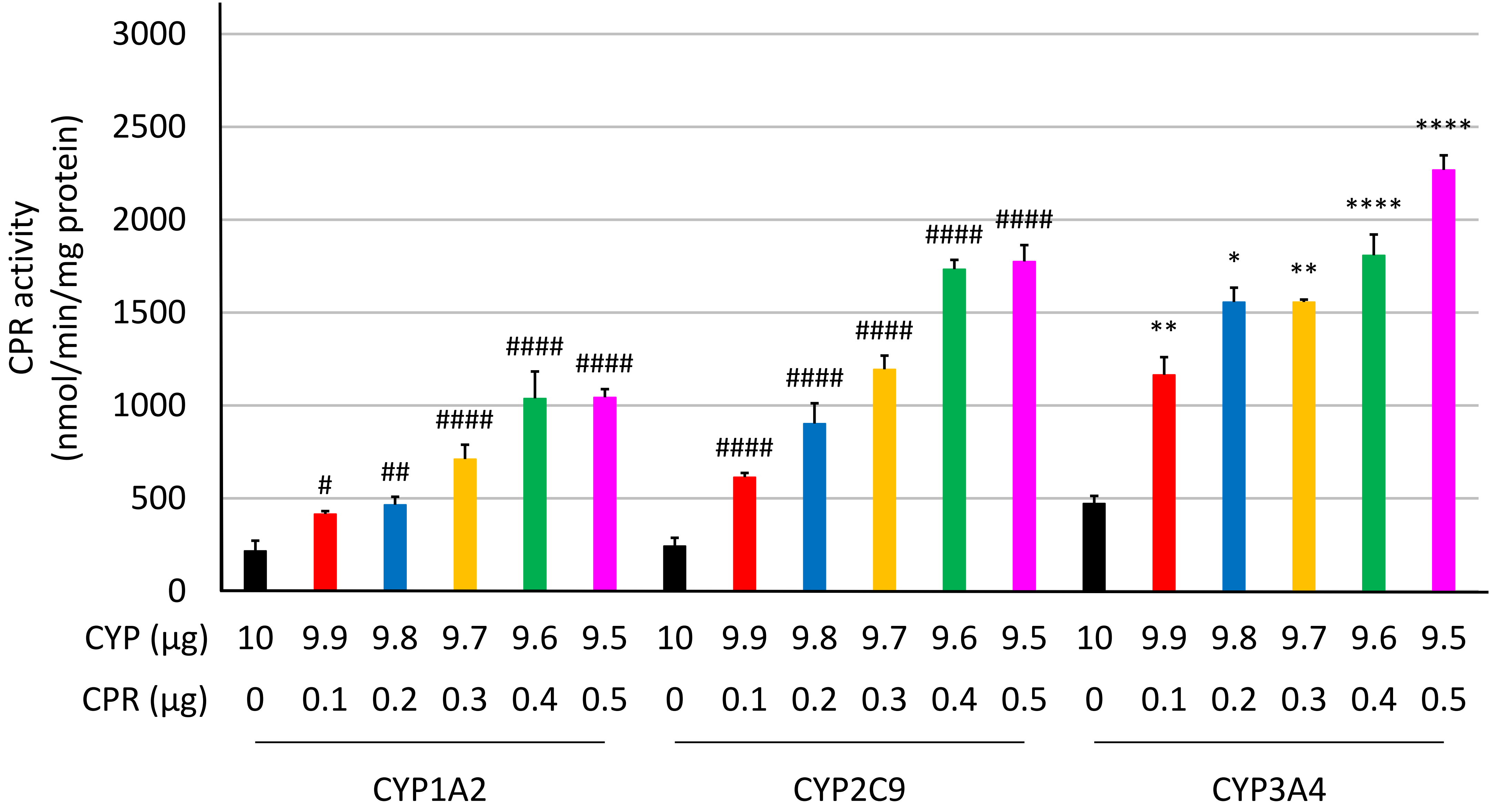

Supplementary Figure 6

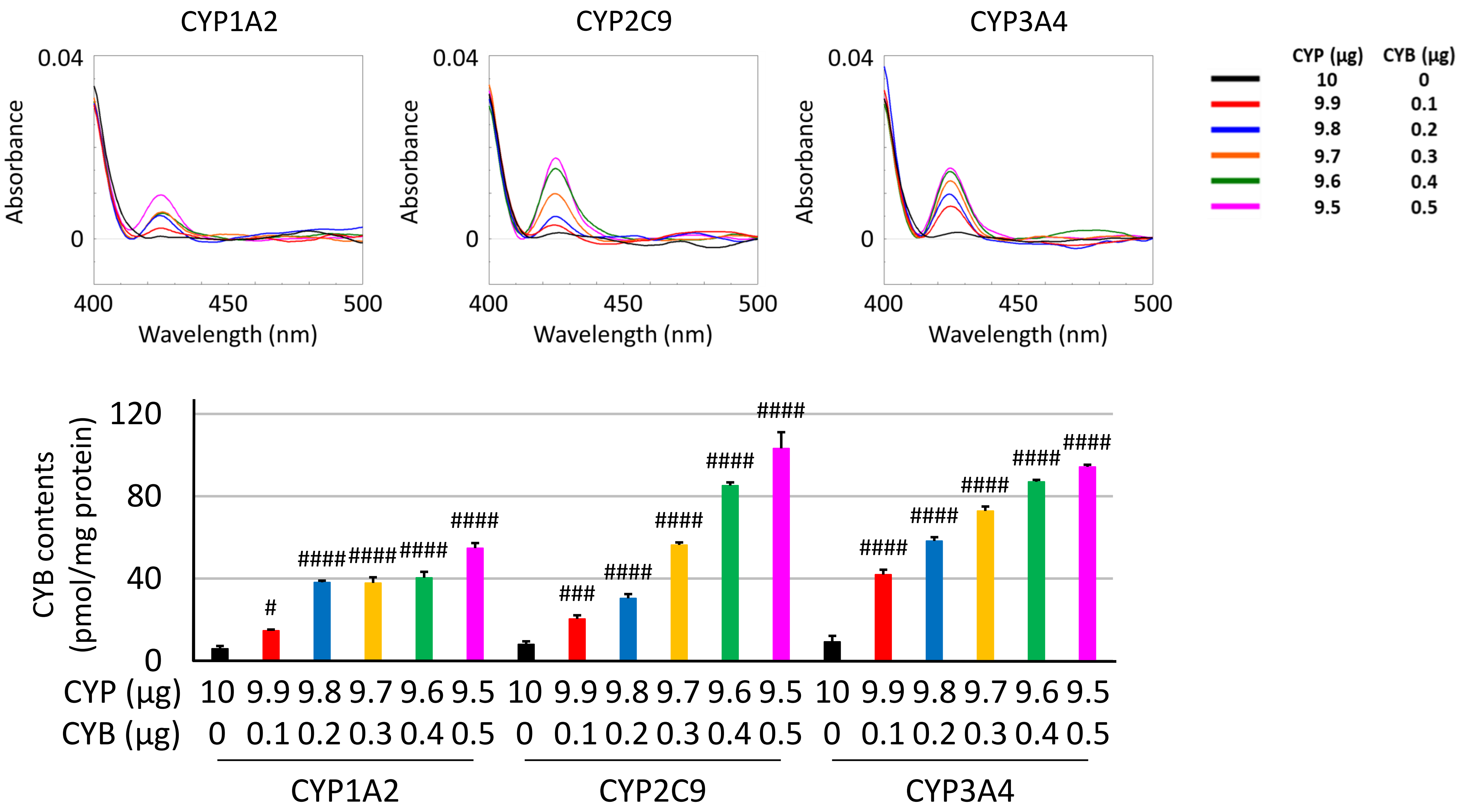

Supplementary Figure 7

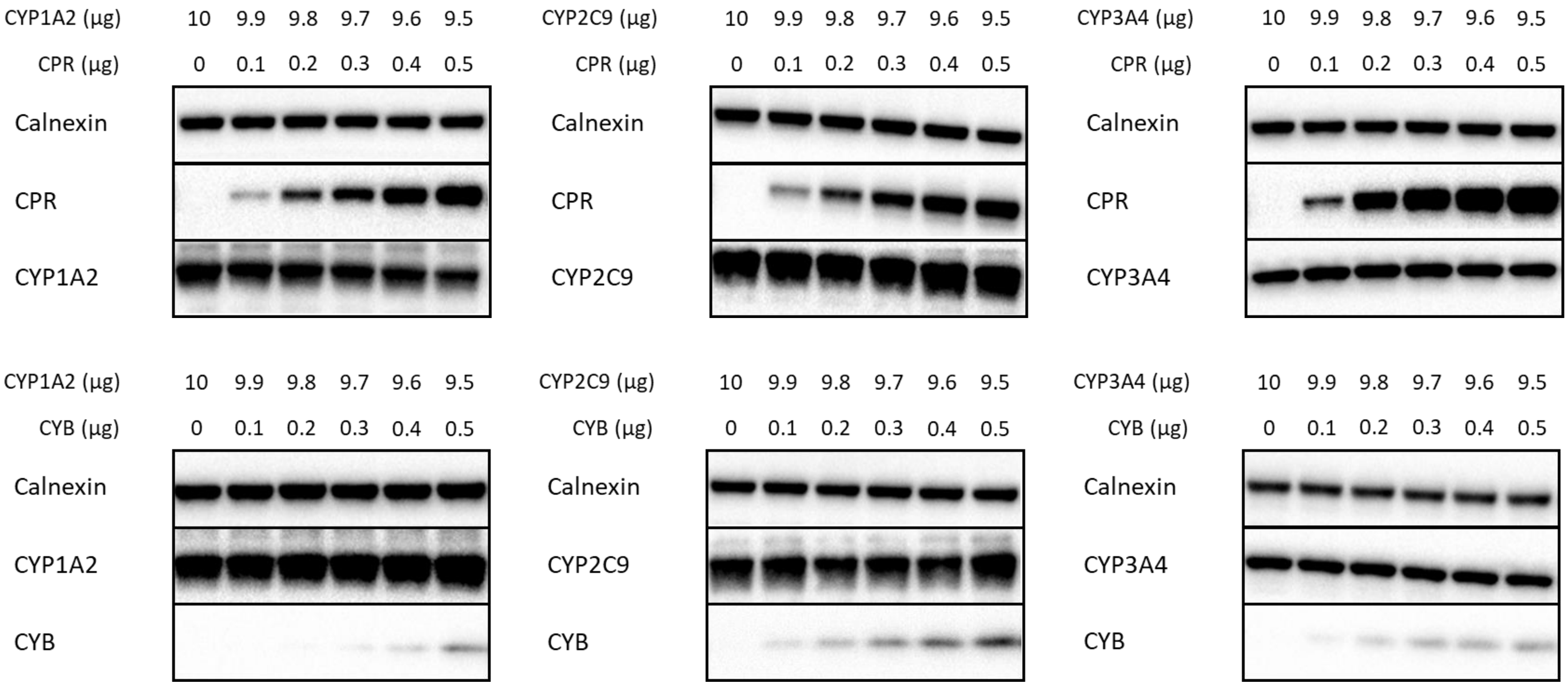

Supplementary Figure 8

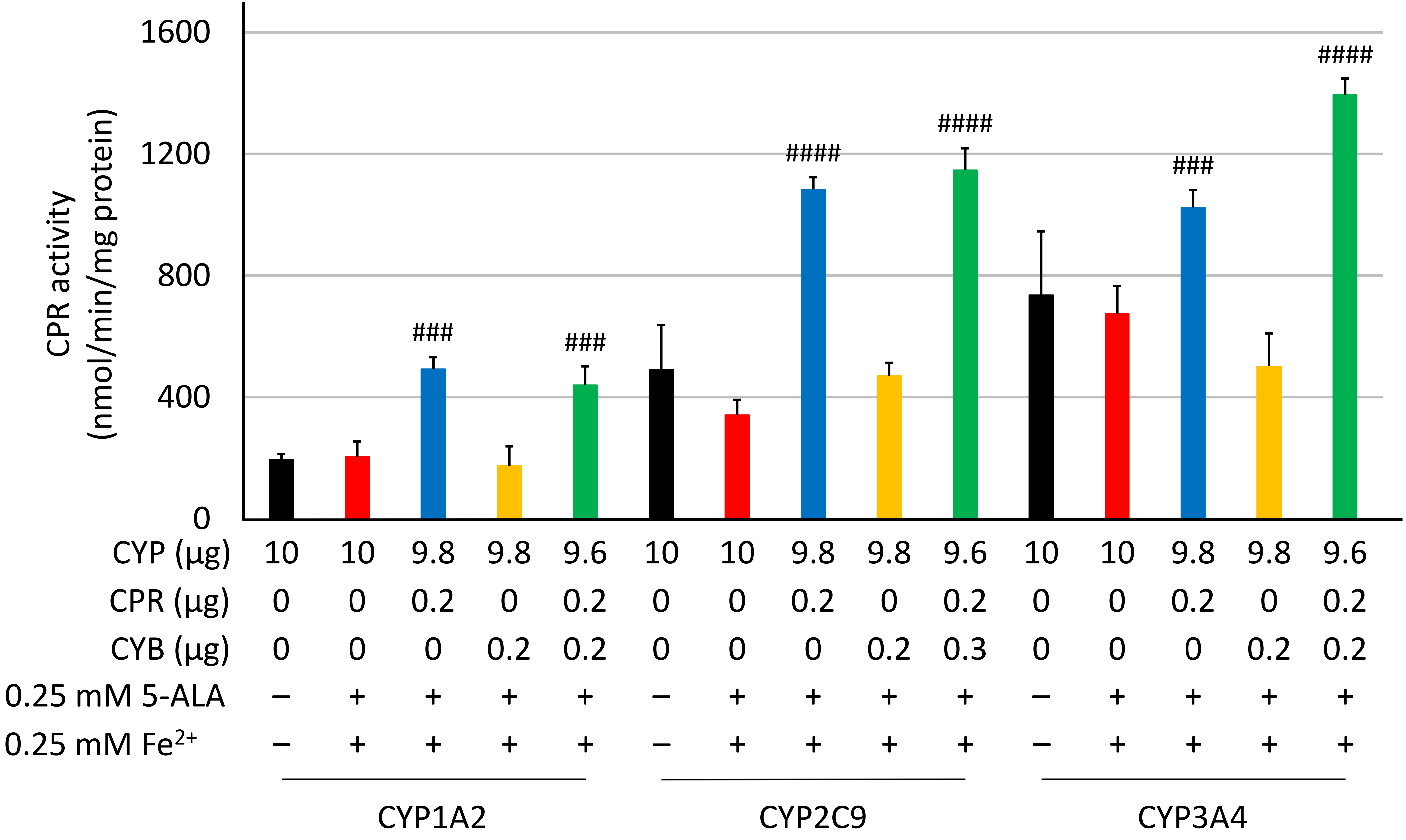

Supplementary Figure 9

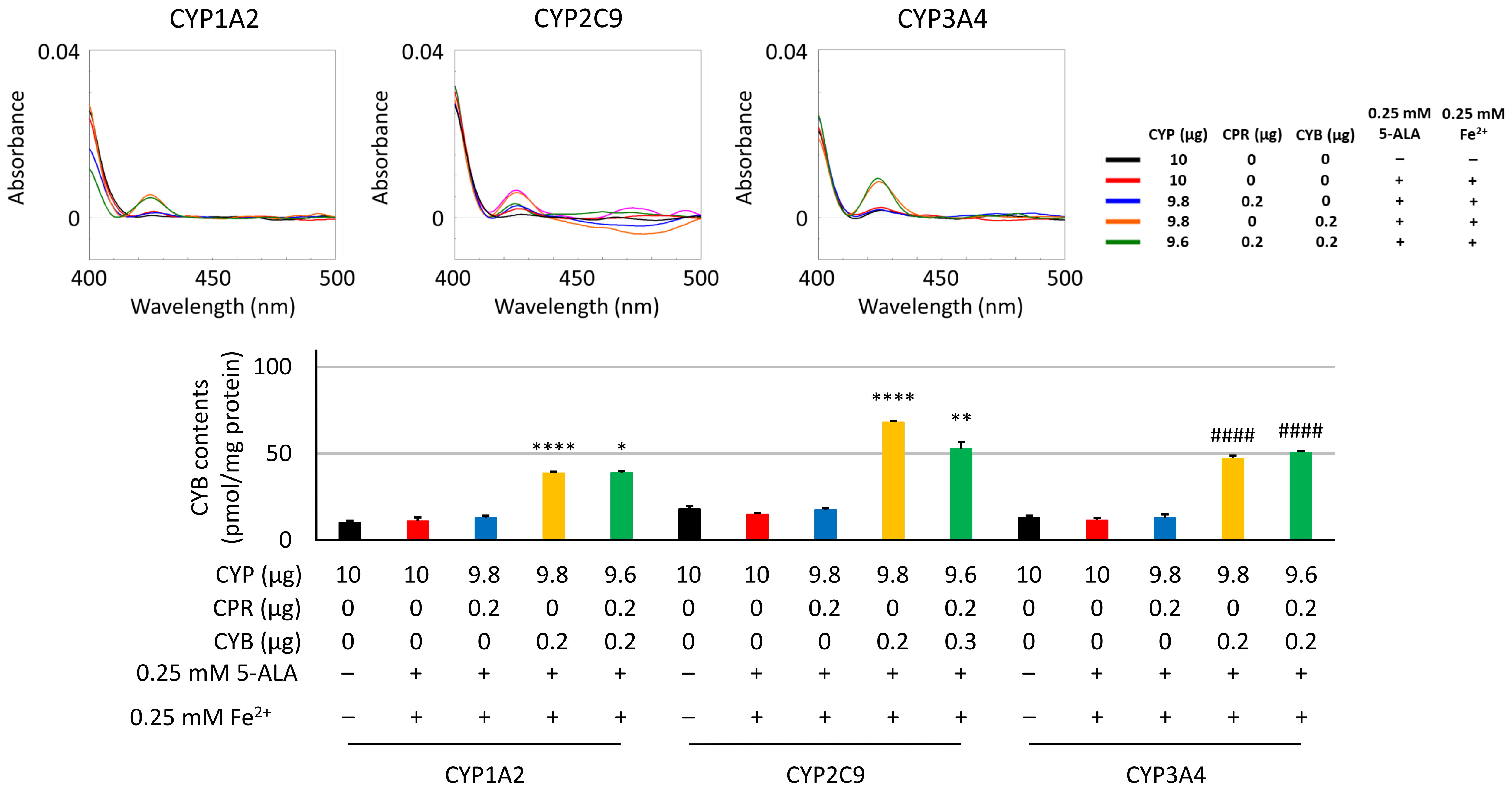

Supplementary Figure 10

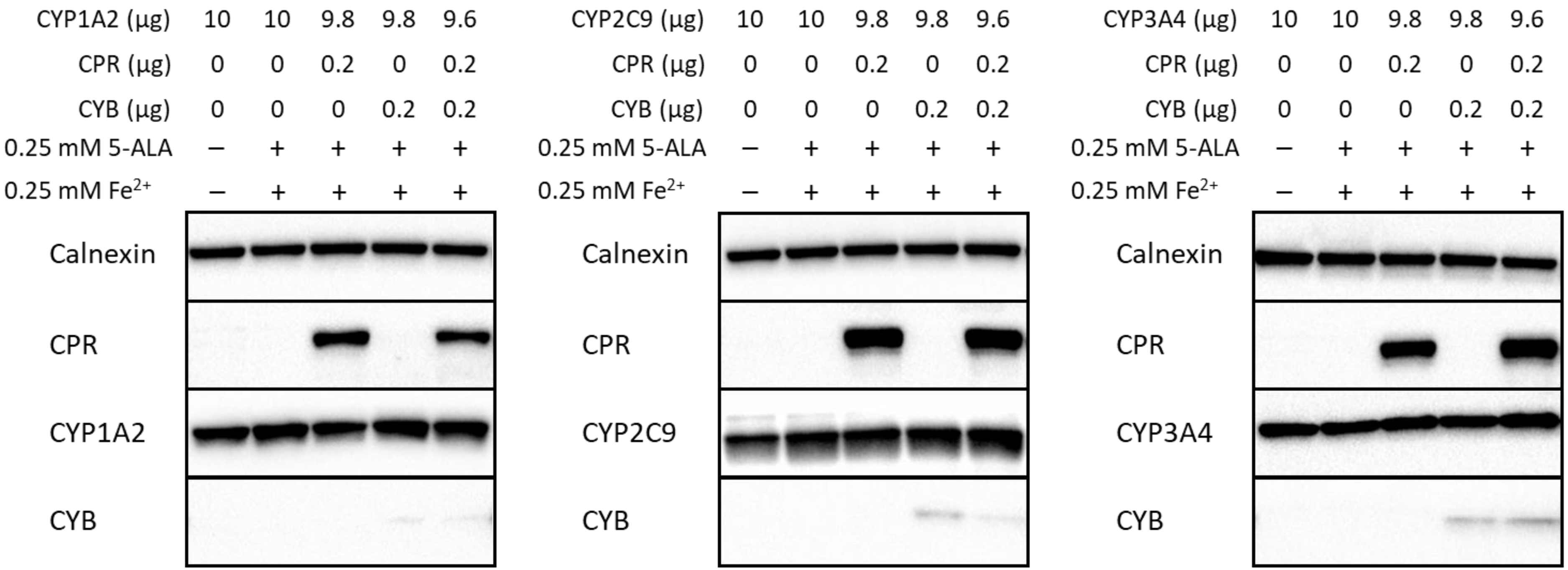

Supplement: Supplementary file 2 — Supplementary figures. [file 41598_2020_71035_MOESM2_ESM.pdf]
