# Supplementary material for: Heterologous expression of high-activity cytochrome P450 in mammalian cells
Source: Sci Rep. 2020 Aug 25;10:14193. doi: 10.1038/s41598-020-71035-5 (PMC7447777; doi:10.1038/s41598-020-71035-5)
Supplement: Supplementary file 3 — Supplementary table 1. [file 41598_2020_71035_MOESM3_ESM.docx]

Supplementary Table 1. List of primary antibodies used in western blotting

| Antigen | Host | Dilution | Source | Cat. No. |
| --- | --- | --- | --- | --- |
| CYP1A2 | Rabbit | 1:5000 | Nosan corporation | PAP021 |
| CYP2C9 | Rabbit | 1:5000 | Nosan corporation | PAP091 |
| CYP3A4 | Rabbit | 1:5000 | Nosan corporation | PAP011 |
| CPR | Rabbit | 1:5000 | Abcam | ab13513 |
| CYB | Rabbit | 1:2000 | Abcam | ab69801 |
| Calnexin | Rabbit | 1:5000 | Enzo Life Technology | ADI-SPA-860 |
